# Supplementary material for: Radiological appearance and lung function six months after invasive ventilation in ICU for COVID-19 pneumonia: An observational follow-up study
Source: PLoS One. 2023 Sep 1;18(9):e0289603. doi: 10.1371/journal.pone.0289603 (PMC10473523; doi:10.1371/journal.pone.0289603)
Supplement: S1 File — (DOCX) [file pone.0289603.s001.docx]

| **Spirometri**  *All patients (n=65)* | **Results**  *Mean (SD)*  *Median (IQR) / (min: max)* |
| --- | --- |
| **Dynamic spirometry, n=65** | |
| FEV1 litres | 2.978 (0.897)  2.970 (1.230) / (1.010: 4.880) |
| FEV1 z-score | -0.447 (1.335)  -0.273 (1.642) / (-3.853: 2.040) |
| FEV1 z-score < -1.96, n (%) | 10 (15) |
| FVC litres | 3.795 (1.205)  3.710 (1.660) / (1.390: 6.480) |
| FVC z-score | -0.585 (1.392)  -0.408 (1.583) / (-4.703: 1.560) |
| FVC z-score < -1.96, n (%) | 9 (14) |
| FEV1/FVC | 0.791 (0.062)  0.796 (0.082) / (0.610: 0.944) |
| FEV1/FVC z-score | 0,173 (0,908)  0,271 (1,123) / (-2,153: 2,733) |
| FEV1/FVC z-score < -1.96, n (%) | 1 (2) |
| **SVC, n = 30** | |
| SVC litres (n = 30) | 4.252 (1.196)  4.310 (1.890) / (1.790: 6.240) |
| SVC z-score (n=30) | -0.914 (1.753)  -0.650 (2.208) / (-5.690: 1.610) |
| SVC z-score < -1.96 (n=30), n (%) | 7 (23) |
| **DLCO, n = 62** | |
| DLCO (mmol/min/kPa) | 6.385 (2.346)  5.975 (2.615) / (2.130: 2.540) |
| DLCO z-score | -1.779 (1.517)  -1.730 (1.727) / (-6.131: 1.091) |
| DLCO z-score < -1.96, n (%) | 27 (44) |
| VA litres | 4.876 (1.425)  4.720 (1.980) / (2.110: 8.140) |
| VA z-score | -1.611 (1.621),  -1.399 (1.900) / (-6.878: 1.053) |
| VA z-score < -1.96, n (%) | 24 (39) |
| DLCO/VA | 1.308 (0.276)  1.275 (0.343) / (0.680: 1.930) |
| DLCO/VA z-score | -0.575 (1.212)  -0.509 (1.482) / (-3.976: 2.063) |
| DLCO/VA z-score < -1.96, n (%) | 7 (11) |
| **VC measurement, n = 33** | |
| VC litres | 3.530 (1.167)  3.350 (1.530) / (1.550: 6.630) |
| VC z-score | -1.156 (1.380)  -1.111 (1.882) / (-4.394: 1.203) |
| VC z-score < -1.96, n (%) | 9 (30) |

*For categorical variables n (%) is presented. For continuous variables, mean (SD) and median (min; max). FEV1 = Forced expiratory reserve volume during the 1^st^ second of expiration; FVC =Forced vital capacity; SVC = Slow vital capacity; DLCO = Diffusion capacity for carbon monoxide; VA = Alveolar volume; VC = Vital capacity; TLC = Total lung capacity; FRC = Functional residual capacity; RV = Residual volume.*

**Table 5**. Supplementary data on lung function presented as absolute and % predicted values as well as z-scores
