# Supplementary material for: Radiological appearance and lung function six months after invasive ventilation in ICU for COVID-19 pneumonia: An observational follow-up study
Source: PLoS One. 2023 Sep 1;18(9):e0289603. doi: 10.1371/journal.pone.0289603 (PMC10473523; doi:10.1371/journal.pone.0289603)
Supplement: Attachment — Submitted filename: Reviewer 1.docx [file pone.0289603.s003.docx]

Reviewer 1

Comment 1 – doesn’t answer my question or state it in the methods

Please add in the methods that radiologists were not blinded and that it was a consensus finding with discussion or whether independent evaluation

Comment 2 sorry didn’t explain properly please list abbreviations in the legend – not all abbreviations are defined in Table 1 eg LOS-ICU BMI etc

Comment 4. Please add these definitions of mild moderate and severe DLCO impairment in the text when the results are presented. Legend isn’t sufficient

Comment 7: As you have the data regarding treatment it is important to present this in the narrative as impacts any conclusions made. Not expected to include in statistical analysis. Please include in results as a sentence
